# Healthcare utilization and unmet needs of patients with antisynthetase syndrome: An international patient survey

**DOI:** 10.1007/s00296-023-05372-9

**Published:** 2023-07-15

**Authors:** M. Weiss, M. T. Holzer, F. Muehlensiepen, Y. Ignatyev, C. Fiehn, J. Bauhammer, J. Schmidt, S. Schlüter, A. Dihkan, D. Scheibner, U. Schneider, L. Valor-Mendez, G. Corte, L. Gupta, H. Chinoy, I. Lundberg, L. Cavagna, J. H. W. Distler, G. Schett, J. Knitza

**Affiliations:** 1 Department of Internal Medicine 3, Friedrich-Alexander-University Erlangen-Nürnberg and Universitätsklinikum Erlangen, Erlangen, Germany; 2 Deutsches Zentrum Immuntherapie (DZI), Friedrich-Alexander-University, Erlangen, Germany; 3 III. Department of Internal Medicine, University Medical Center Hamburg-Eppendorf, Hamburg, Germany; 4 Faculty of Health Sciences, Center for Health Services Research, Brandenburg Medical School Theodor Fontane, Rüdersdorf, Germany; 5 Praxis für Rheumatologie, Klinische Immunologie, Medical Center, Baden-Baden, Germany; 6 Faculty of Health Sciences Brandenburg, Brandenburg Medical School Theodor Fontane, Rüdersdorf bei Berlin, Germany; 7 Department of Neurology and Pain Treatment, Center for Translational Medicine, Neuromuscular Center, Immanuel Klinik Rüdersdorf, University Hospital of the Brandenburg Medical School Theodor Fontane, Rüdersdorf bei Berlin, Germany; 8 Department of Neurology, Neuromuscular Center, University Medical Center, Göttingen, Germany; 9 Myositis-Gruppe, Deutsche Gesellschaft Für Muskelkranke, Freiburg, Germany; 10 The Swedish Working Group for Myositis, The Swedish Rheumatism Association, Stockholm, Sweden; 11 Department of Rheumatology, Charité - Universitätsmedizin Berlin, Corporate Member of Freie Universität Berlin and Humboldt-Universität zu Berlin, Berlin, Germany; 12 Department of Rheumatology, Royal Wolverhampton Hospitals NHS Trust, Wolverhampton, UK; 13 Division of Musculoskeletal and Dermatological Sciences, School of Biological Sciences, Centre for Musculoskeletal Research, The University of Manchester, Manchester, UK; 14 Department of Rheumatology, City Hospital, Sandwell and West Birmingham Hospitals NHS Trust, Birmingham, UK; 15 Department of Rheumatology, Salford Royal Hospital, Northern Care Alliance NHS Foundation Trust, Manchester Academic Health Science Centre, Salford, UK; 16 Division of Musculoskeletal and Dermatological Sciences, Faculty of Biology, Medicine and Health, The University of Manchester, Manchester, UK; 17 Division of Rheumatology, Department of Medicine, Karolinska Institutet, Karolinska University Hospital, Solna, Stockholm Sweden; 18 Department of Gastroenterology, Dermatology and Rheumatology, Karolinska University Hospital, Stockholm, Sweden; 19 Rheumatology Division, Fondazione Istituti di Ricovero e Cura a Carattere Scientifico (IRCCS) Policlinico San Matteo, Pavia, Italy

**Keywords:** Myositis, Autoantibodies, Health services research, Therapeutics, Diagnosis

## Abstract

Antisynthease syndrome (ASSD) is a rare, complex and understudied autoimmune disease. Internet-based studies can overcome barriers of traditional on-site research and are therefore very appealing for rare diseases. The aim of this study was to investigate patient-reported symptoms, diagnostic delay, symptoms, medical care, health status, working status, disease knowledge and willingness to participate in research of ASSD patients by conducting an international web-based survey. The multilingual questionnaire was created by an international group of rheumatologists and patients and distributed online. 236 participants from 22 countries completed the survey. 184/236 (78.0%) were female, mean age (SD) was 49.6 years (11.3) and most common antisynthetase antibody was Jo-1 (169/236, 71.6%). 79/236 (33.5%) reported to work full-time. Median diagnostic delay was one year. The most common symptom at disease onset was fatigue 159/236 (67.4%), followed by myalgia 130/236 (55.1%). The complete triad of myositis, arthritis and lung involvement verified by a clinician was present in 42/236 (17.8%) at disease onset and in 88/236 (37.3%) during the disease course. 36/236 (15.3%) reported to have been diagnosed with fibromyalgia and 40/236 (16.3%) with depression. The most reported immunosuppressive treatments were oral corticosteroids 179/236 (75.9%), followed by rituximab 85/236 (36.0%). 73/236 (30.9%) had received physiotherapy treatment. 71/236 (30.1%) reported to know useful online information sources related to ASSD. 223/236 (94.5%) were willing to share health data for research purposes once a year. Our results reiterate that internet-based research is invaluable for cooperating with patients to foster knowledge in rare diseases.

**Supplementary Information:**

The online version contains supplementary material available at 10.1007/s00296-023-05372-9.

## Introduction

Antisynthetase-syndrome (ASSD) as one of the idiopathic inflammatory myopathy (IIM) subtypes is a rare disease, with an estimated global prevalence of 1-9/100.000 [1]. In an analysis within the EuroMyositis registry, ASSD was the third most common IIM subtype (17% of analyzed patients) after dermatomyositis and polymyositis [2]. The defining diagnostic cornerstone is the presence of autoantibodies against one of the aminoacyl transfer RNA (tRNA) synthetases. Anti-Jo1 auto-antibodies were the first described antibodies of this disease spectrum [3]. Furthermore, they are the only myositis specific autoantibodies used in the latest EULAR/ACR criteria for IIM [4]. Other antibodies defining ASSD are anti-PL-7, anti-PL-12, anti-EJ, anti-OJ, anti-KS, anti-Ha and anti-Zo [5]. In addition to muscle involvement, the main clinical features are Raynaud’s phenomenon, mechanic’s hands, arthritis, and interstitial lung disease (ILD) [5]. Although classified as IIM, myositis might be clinically absent in some cases [6] and symptoms vary remarkably between patients [7]. Prognosis mainly depends on pulmonary involvement, often seen in anti-PL-7/-12 patients [8]. Therapy is established depending on organ involvement [9] and mainly includes methotrexate, azathioprine, cyclophosphamide, rituximab or IVIG [10]. Especially in ASSD with concomitant ILD, B-cell-directed therapy with rituximab is increasingly used [11] and was associated with a significant glucocorticoid-sparing effect [12].

Digital crowdsourcing promises to overcome barriers of traditional on-site research [13]. The validity of patient-reported diagnoses has been underlined in previous publications [14–16] and lead to first patient-powered, internet-based studies in rheumatology [17–20]. Despite the increasing research regarding ASSD in the last few years, aspects such as disease knowledge, quality of life, burden of disease, healthcare utilization and willingness to participate in research remained largely unexplored. Elucidating these questions may help to improve the health care situation of ASSD patients.

The aim of this study was to investigate patient-reported symptoms, diagnostic delay, medical care, health and working status, disease knowledge and willingness to participate in research of ASSD patients by conducting an international web-based survey.

## Materials and methods

### Survey development

The survey was initially drafted in German by JK and DS and was then adopted to the suggestions of all authors including rheumatologists treating ASSD patients and three patients’ representatives to ensure meaningfulness and clarity of the questions asked. The questionnaire was translated to five additional languages (English, Swedish, Spanish, French, Italian) by native speakers to guarantee equivalence. The final multilingual online questionnaire (Supplementary Material 1) included 4 general questions and 33 different items and took approximately 10 min to complete. The group decided to investigate: (1) Symptoms and diagnostic delay – 9 items (2) medical care – 9 items (3) health and working status – 7 items (4) disease knowledge – 6 items and (5) research participation – 2 items.

### Participant recruitment

The cross-sectional questionnaire (SurveyMonkey Inc) was available in six languages (English, German, Swedish, Spanish, French, Italian) and accessible from 4th of July 2020 until 23rd April 2021. No specific sample size was targeted. The survey was distributed via social media (Twitter, Instagram, Facebook), QR codes and email. All patients with self-reported ASSD diagnosis confirmed by a physician were eligible. This survey was based on patient reported aspects, as the questions were answered anonymously the gained information based solely on patients’ answers and not on information of the treating physician. Prior to completing the survey patients were required to give consent via the platform.

### Statistical analysis

Descriptive and summary statistics were used and depicted in tabular and graphical form using mean, median, percentage or minimum/maximum to enable comparison e.g. between ASSD subtypes. Participants, who did not complete the whole survey were excluded from statistics. Regression analyses were performed to investigate correlations. The analyses were performed using IBM SPSS Statistics V.22 Windows (SPSS Inc, Chicago, Illinois, USA). Results and methodology was reported according to the Checklist for Reporting Results of Internet E-Surveys [21]. Graphs were depicted with Microsoft Excel. The study was approved by the ethics committee of the medical faculty of the university of Erlangen-Nürnberg, Germany (20-104-B; date: 16.03.2020).

## Results

### Participant characteristics

337 participants started the survey, of which 236/337 (70.0%) completed the survey. 91/236 (38.6%) and 26/236 (11.0%) found out about the survey from a patient community and a treating physician, respectively. 184/236 (78.0%) were female, mean age (SD) was 49.6 years (11.3 years), see Table 1. Patients from a total of 22 countries participated in this study. Most common country of residency was Germany (82/236, 34.8%) followed by USA (38/236, 16.1%) and UK (28/236, 11.9%). Most common antisynthetase antibody was Jo-1 (169/236, 71.61%). 27.5% (65/236) of participants smoked in the past and 4.7% (11/236) reported to be currently smoking. Patients additionally reported to have been diagnosed with fibromyalgia (36/236, 15.3%), depression (40/236, 16.3%) or a malignancy (5/236, 2.1%).

**Table 1:** Patient demographics

Patients Total	n = 236
Female, n (%)	184 (78.0%)
Age, years, mean ± SD	49.6 ± 11.4
Age at diagnosis, years, mean ± SD	43.6 ± 12.2
Disease duration^**a**^, years, median (IQR) Disease duration^**a**^, years, mean ± SD	4.0 (2.0–7.3) 5.9 ± 6.2
Diagnostic delay^b^, years, median (IQR) Diagnostic delay^b^, years, mean ± SD	(0.0–2.0) 2.2 ± 4.4
Number of specialist physicians seen until diagnosis, median (IQR)	3.0 (2.0–4.0)
Smoking status	
Non smoking, n (%)	160 (67.8%)
Currently, n (%)	11 (4.7%)
Only in the past, n (%)	65 (27.5%)
Antisynthetase antibody	
Anti-Jo-1, n (%)	169 (71.6%)
Anti-PL-7, n (%)	33 (14.0%)
Anti-PL-12, n (%)	34 (10.2%)
Anti-EJ, n (%)	5 (2.1%)
Anti-OJ, n (%)	4 (1.7%)
Other, n (%)	34 (14.4%)
Country of residence	
Germany, n (%)	82 (34.7%)
USA, n (%)	37 (15.7%)
United Kingdom, n (%)	28 (11.9%)
Sweden, n (%)	17 (7.2%)
Italy, n (%)	12 (5.1%)
France, n (%)	10 (4.2%)
Spain, n (%)	3 (1.3%)
Switzerland, n (%)	2 (0.9%)
Austria, n (%)	2 (0.9%)
Other, n (%)	43 (18.2%)

^a^ Time between symptom onset and survey completion

^b^ Time between symptom onset and diagnosis

### Diagnostic delay

Mean age (SD) at diagnosis was 49.5 years (11.3 years) with a median (IQR) diagnostic delay of 1 year (0–2 years). Maximal diagnostic delay was 31 years. A median (IQR) of 3 (2–4) and a maximum of 30 specialist doctors were seen until the diagnosis ASSD was established. Diagnostic delay for patients with EJ-antibodies was shortest and PL-7-antibodies longest with a median of 0 years (0–1 years) and 2 years (0–5 years), respectively.

### Disease symptoms at onset

**Fig. 1:**
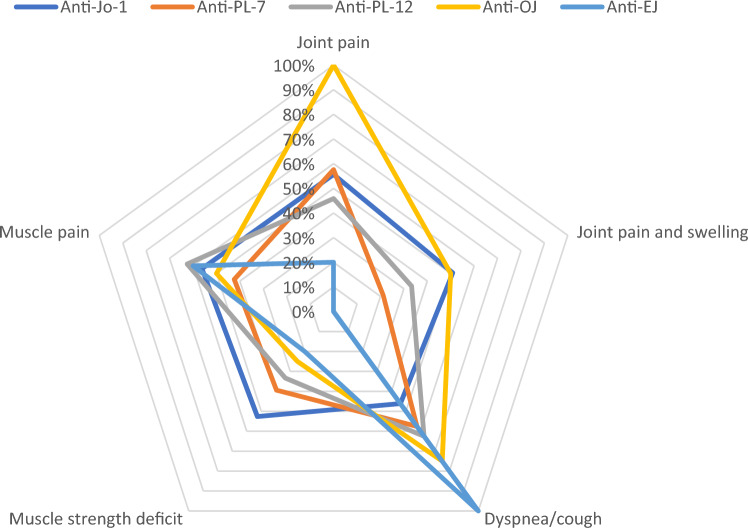
Spider diagram displaying symptoms at disease onset according to antisynthetase antibody status

**Fig. 2:**
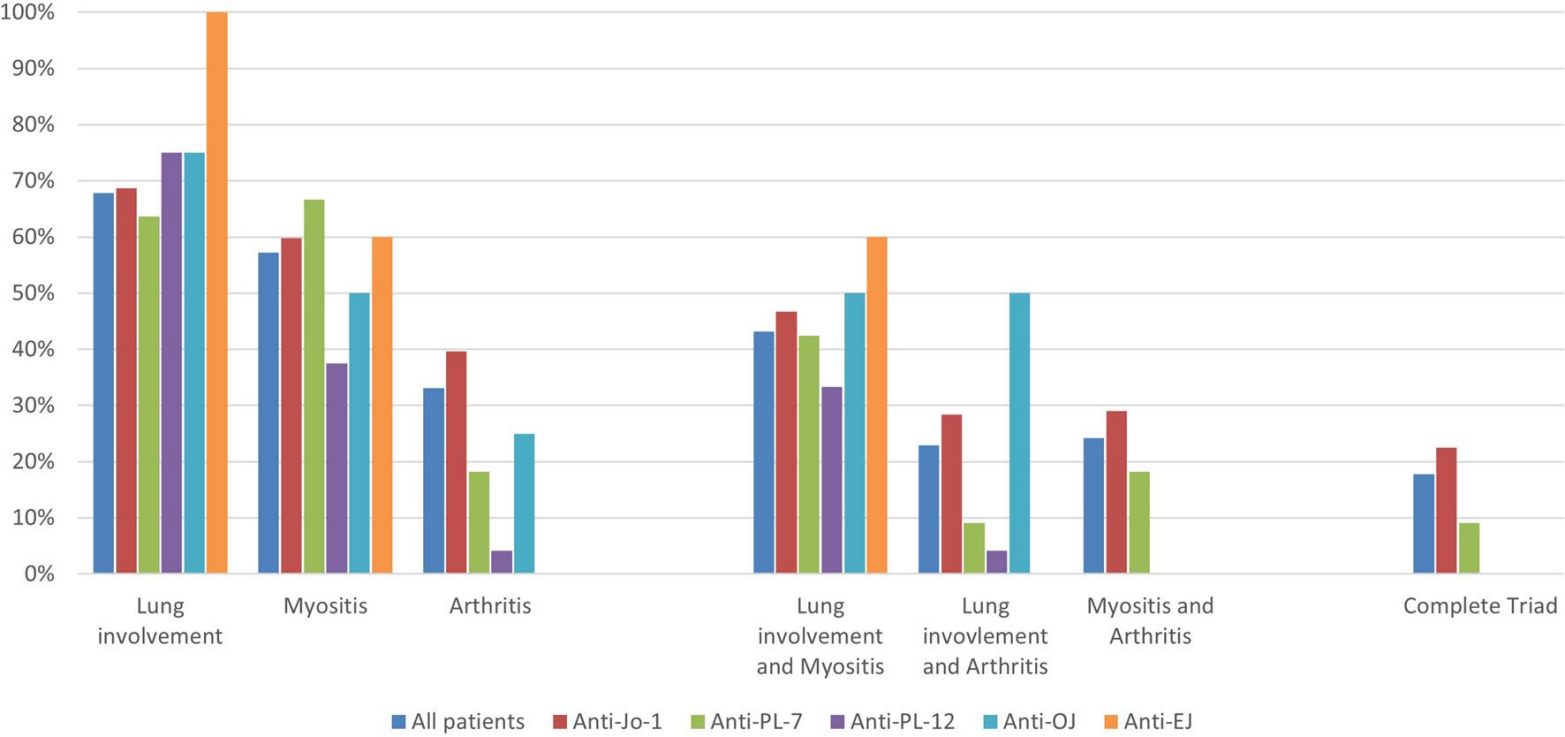
Physician confirmed lung involvement, myositis and arthritis according to antibody status at disease onset

Most common patient reported symptoms at disease onset were fatigue (159/236, 67.4%), followed by muscle pain (130/236, 55.1%) and joint pain (125/236, 53.0%) see Fig. 1 and supplementary table 1. Patients reported clinician verified myositis, arthritis and interstitial lung disease at disease onset in 57.2% (135/236), 33.1% (78/236), 67.8% (160/236), respectively, see Fig. 2 and supplementary table 2. The complete triad of myositis, arthritis and lung involvement verified by a clinician was present in 17.8% (42/236) at disease onset and in 37.3% (88/236) during the disease course of the patients.

### Medical care

**Fig. 3:**
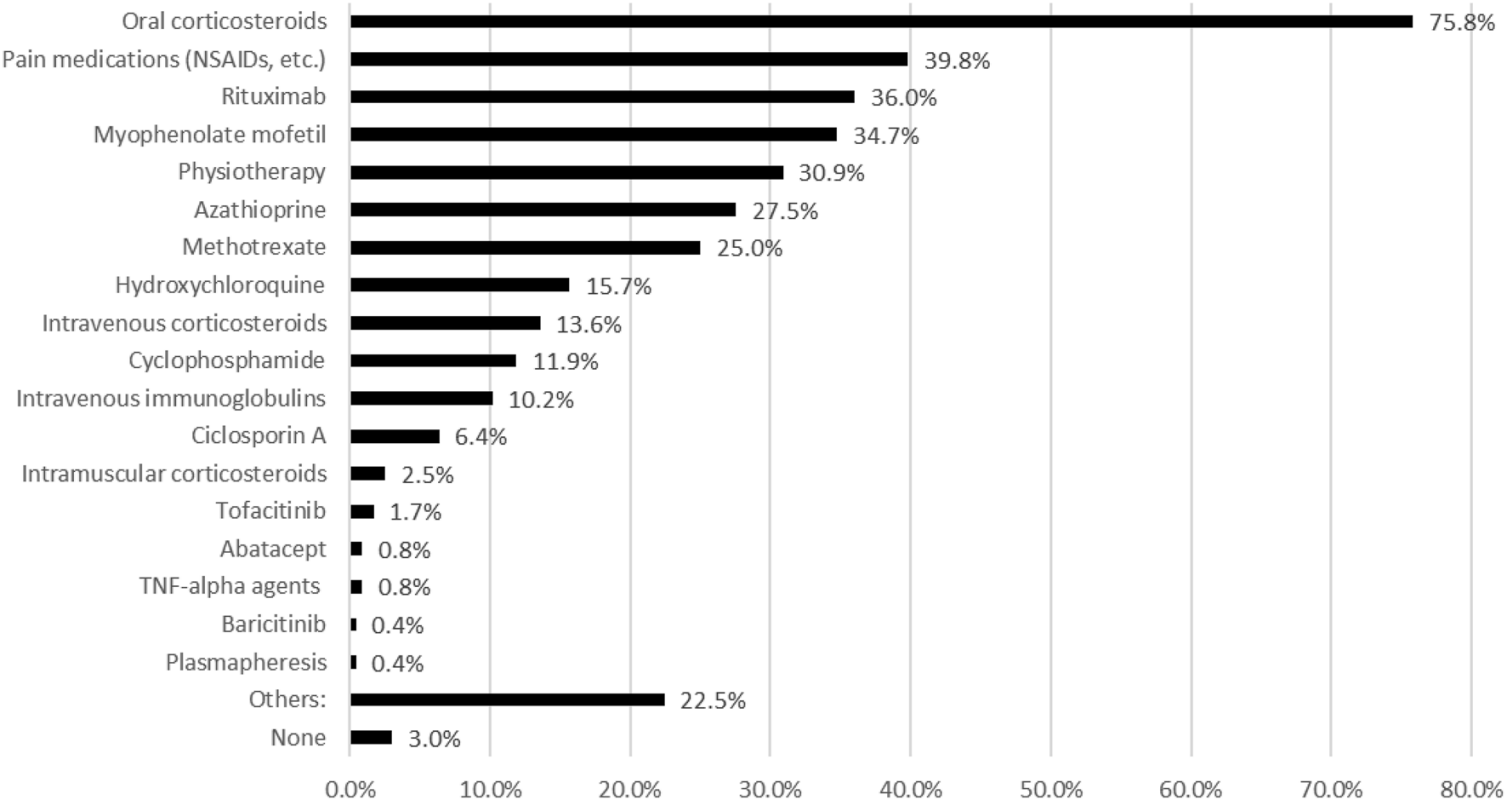
Current and previous treatments

Patients reported a rheumatologist most often as their main physician contact person (187/236, 79.2%) (supplementary table 3) for ASSD and reported to have seen this physician on average (SD) 4.8 times (4.5) during the last year. 46.6% (110/236) of these main physician contacts worked at a university center. Most patients were regularly seen by a rheumatologist (216/236, 91.5%) followed by a pulmonologist (127/236, 53.8%), see supplementary table 4. 153/236 (64.8%) were regularly seen by at least two specialists, most often by a rheumatologist and a pulmonologist (140/153, 91.5% and 124/153, 81.0%). Table 2 displays the tests reported to have been carried out to diagnose and evaluate ASSD. Most often conducted for diagnosis was a pulmonary function test (198/236, 83.9%), followed by a computer tomography (166/236, 70.3%). Most commonly reported immunosuppressive treatment were oral corticosteroids (179/236, 75.9%) followed by rituximab (85/236, 36.0%), see Fig. 3.

**Table 2:** Reported tests carried out to establish and evaluate diagnosis

Reported tests, n (%)	Total n = 964
Pulmonary function test	198 (84.0%)
Computer tomography scan	166 (70.3%)
Magnetic resonance imaging	116 (49.2%)
Muscle strength test	92 (39.0%)
Muscle biopsy	86 (36.4%)
Electromyography	83 (35.2%)
Joint X-rays	79 (33.3%)
Joint ultrasound	38 (16.1%)
Nailfold videocapillaroscopy	38 (16.1%)
Muscle endurance test	35 (14.4%)
Muscle ultrasound	33 (14.0%)

### Health and working status

98/236 (41.5%) patients rated their general health status as fair, 4/236 (1.7%) as excellent, 23/236 (9.8%) as very good, 74/236 (31.4%) as good and 47/236 (15.7%) as poor, respectively. Compared to one year ago, 67/236 (28.4%) reported that their health is about the same now. 79/236 (33.5%) and 28/236 (11.9%) reported to work full-time or part-time, respectively and 29/236 (12.3%) and 16 (6.8%) reported inability to work or currently being on sick leave, respectively. Mean days of sick leave during the last year were on average (SD) 46.2 (100.1).

### Disease knowledge

**Fig. 4:**
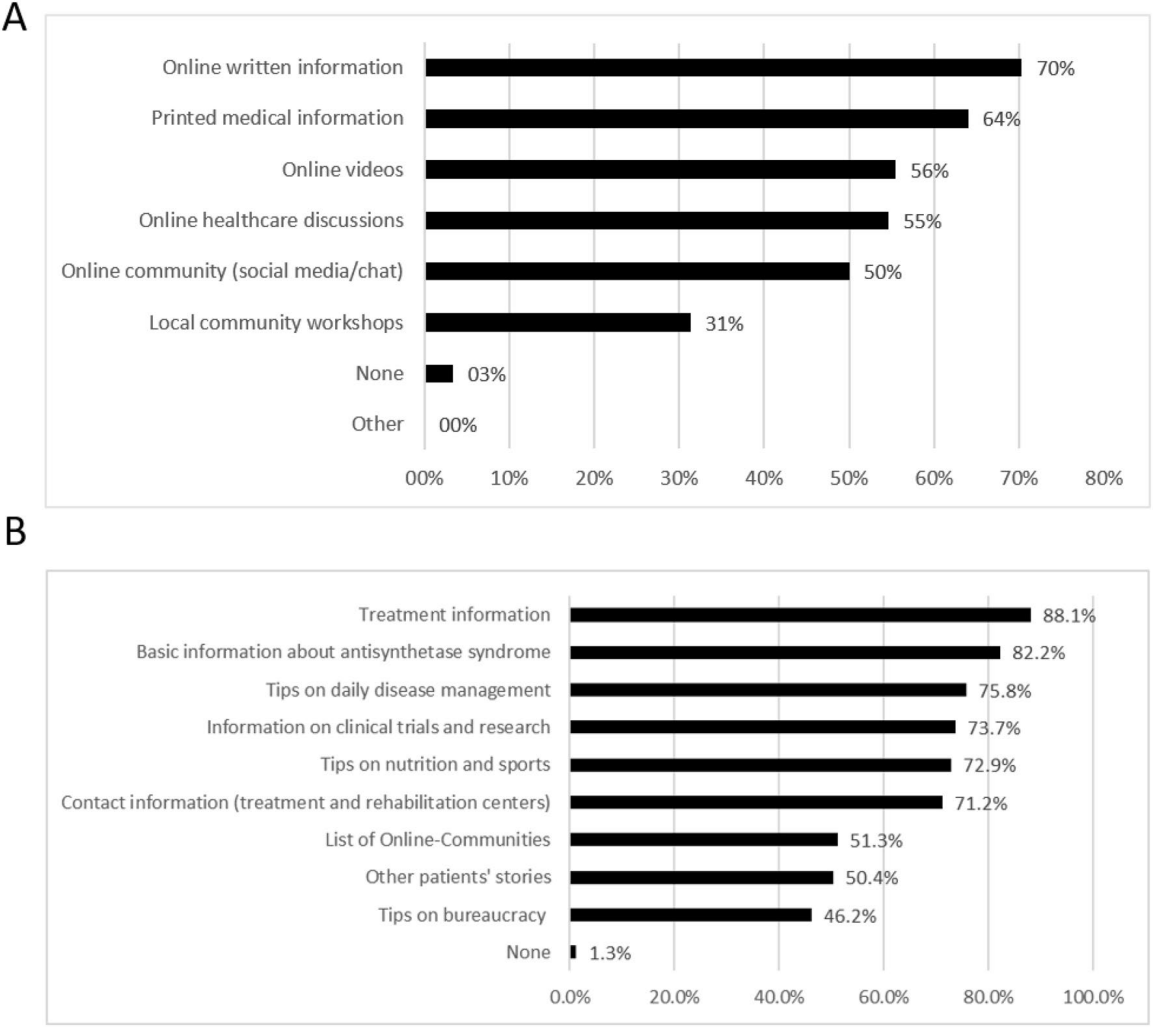
Patient preferences regarding information format (**A**) and information topics (**B**)

167/236 (70.8%) and 142/236 (60.2%) of patients rated their disease knowledge and knowledge about treatment options as good (instead of poor), respectively. 71/236 (30.1%) reported to know useful online information sources related to ASSD. Supplementary table 5 lists all links that were reported as useful by patients. 227/236 (96.2%) were interested in additional disease related information. Regarding the information format, patients expressed greatest interest in online written information (166/236, 70.3%) and least interest in local community workshops (73/236, 31.4%), see Fig. 4A. Information regarding treatment information was most desired by patients (208/236, 88.1%), see Fig. 4B.

### Research participation

223/236 (94.5%) of the patients reported to be willing to regularly share data about their health for research purposes once a year. Patients were willing to share data about symptoms (216/236, 91.5%), life quality (205/236, 68.9%), blood test results (163/236, 69.1%), provide actual blood samples (114/236, 48.3%), data from wearables (78/236, 33.1%).

## Discussion

To our knowledge, this is the first large international survey among patients with a self-reported diagnosis of ASSD. The results depict the current healthcare situation of ASSD patients, pointing out unmet needs and may guide further research. Furthermore, this web-based study underlines how technology may facilitate and accelerate research in rare diseases.

The median diagnostic delay was similar to the previous study of Cavagna et al. with one year, and anti-PL-7/12 patients reporting longer delays compared to anti-Jo-1 patients [7]. The recently published systematic review on diagnostic delay of myositis patients revealed a mean delay of 17 months in diagnosis of ASSD with very varying study results [22]. As possible reasons for the delay in diagnosis many factors like health care service-related aspects or complex clinical characteristics were discussed. Classification criteria for ASSD are currently being developed in a collaborative project (CLASS) to further improve collaborative research but also aid diagnosis. Levi et al. demonstrated the value of a multidisciplinary team and especially including rheumatologists to increase diagnostic accuracy [23].

In this study the large majority of patients (91.5%) was seen regularly by rheumatologists, however this could be due to selection bias, as rheumatologists mainly helped to share the survey. Interestingly, less than half of patients (46.6%) reported to have a main physician contact working at a university center. This could also partly explain the relatively low number of tests reported to establish the diagnosis (i.e. 39.0% reporting a muscle strength test). A recent survey among physicians treating IIM patients revealed the inconsistent state of knowledge and clinical practice [24].

In line with previous studies [17, 25, 26], we observed fatigue (60.7%) and pain (33.5%) being frequently reported. These symptoms should be frequently assessed and could be added to the currently used IMACS Disease Activity Core Set Measures [27]. The major effect of fatigue on overall quality of life of myositis patients has been recently pointed out [28]. Additionally, in our survey 16.3% of the patients described depression as comorbidity. A recent German study of health insurance data found comparable results with slightly elevated depression rates in myositis patients compared to the control group [29]. Hence, fatigue and depression should be actively screened for by physicians in clinical routine.

It is important to note, that lack of physical activity comprises a higher risk of depression [30]. On the other hand, there are studies pointing out the potential effect of physical therapy on pain and functional outcome in myositis patients [31]. Bhashyam et al. recently described the high percentage of opioid users among myositis patients [17]. Noteworthy, our results show higher frequency of pain medication therapy than physiotherapy in ASSD patients. Given the abovementioned aspects of physical activity, there might be an unmet need to improve especially physiotherapy and physical activity in ASSD. The recently published letter by Gupta et al. addresses this need and suggests online solutions and tools to improve self-managed physical activity [32].

Regarding the clinical symptoms we observed highest involvement of joints in Jo-1-positive patients which is congruent to the findings of Cavagna et al. [7]. The complete triad of symptoms was also mainly present in Jo-1-positive, also confirming the results of Cavagna et al. Regarding lung involvement differences between our study and the study of Cavagna et al. can be found. All of the EJ-positive patients had physician proven ILD, which reflects also the initial symptoms of dyspnoe/cough mentioned by the patients. Overall lung involvement in our study (67.8%) confirms the high ILD frequency in ASSD, also observed in the EuroMyositis registry (71%) [2].

In our cohort, rituximab was the most applied therapy, except for oral prednisolone and pain medication (36% of the patients). The increase of this therapeutic concept may depict the latest achievements in immunology, suggesting a potential pathogenic role of ASSD-autoantibodies such as Jo-1 in pathogenesis of the disease [33–35] and therefore giving underlining indications for B-cell-directed treatment approaches in ASSD. Recently a first case of CD-19-targeted CAR T Cells was published demonstrating a novel effective and safe treatment option for refractory cases [36].

The impaired health status of the majority of patients is in line with previous work [37] and prevented the majority of patients from working. Only one third of the patients were able to work full-time. These results confirm the Swedish results by Leclair et al. [38], which demonstrated that costs related to IIM start to increase already two years before diagnosis and mean annual costs were 3 to 5 times higher than in the general population. A German study showed that sick leave and early retirement decreased in the last decades [25].

Patients reported a very high willingness to participate in research activities. Simple patient-centered tests to measure muscle endurance [39] and self-sampling of capillary blood [40] have recently been validated. They could complement wearable [41] and smartphone data [42] to enable remote monitoring [43] and decentralized clinical trials.

Similar to previous studies [19, 44, 45], patients expressed interest for further information and only a third reported to know useful online information regarding ASSD. Reported useful online material was collected (supplementary table 5) and should be proactively discussed especially with newly diagnosed patients. Low interest in local community workshops could be explained by the large burden to participate in such meetings and have been increasingly carried out virtually due to the COVID pandemic.

The large number of international patients and involvement of patient research partners are strengths of this study. The digital nature of the study, recall bias, lack of ability to confirm disease and focus on rheumatologists could introduce a selection bias. Furthermore, not having differentiated between current and previous treatments limit the results. The survey was only evaluated by rheumatologists and the three patient research partners prior to distribution.

## Conclusion

This survey enabled a detailed overview of the current healthcare situation of ASSD patients and unmet needs. The study highlights the great burden of disease, its heterogenous appearance and treatments. The results prompt physicians to evaluate currently neglected symptoms such as fatigue and myalgia and make more usage of physiotherapy. Online information is welcomed by patients however patients are mostly not aware of useful content. The large majority of patients would be willing to share health related data for research purposes. Our results reiterate that internet-based research is invaluable for cooperating with patients to foster knowledge in rare diseases.

## Supplementary Information

Below is the link to the electronic supplementary material.

Supplementary file1 (DOCX 39 KB)

Supplementary file2 (DOCX 20 KB)

## Data Availability

Data are available on reasonable request from the corresponding author.

## References

[1] Opinc AH, Brzezińska OE, Makowska JS (2019). Disability in idiopathic inflammatory myopathies: questionnaire-based study. Rheumatol Int.

[2] Lilleker JB, Vencovsky J, Wang G (2018). The EuroMyositis registry: an international collaborative tool to facilitate myositis research. Ann Rheum Dis.

[3] Hochberg MC, Feldman D, Stevens MB (1984). Antibody to Jo-1 in polymyositis/dermatomyositis: association with interstitial pulmonary disease. J Rheumatol.

[4] Lundberg IE, Tjärnlund A, Bottai M (2017). 2017 European league against rheumatism/American college of rheumatology classification criteria for adult and juvenile idiopathic inflammatory myopathies and their major subgroups. Ann Rheum Dis.

[5] Zanframundo G, Faghihi-Kashani S, Scirè CA (2022). Defining anti-synthetase syndrome: a systematic literature review. Clin Exp Rheumatol.

[6] Friedman AW, Targoff IN, Arnett FC (1996). Interstitial lung disease with autoantibodies against aminoacyl-tRNA synthetases in the absence of clinically apparent myositis. Semin Arthritis Rheum.

[7] Cavagna L, Trallero-Araguás E, Meloni F (2019). Influence of antisynthetase antibodies specificities on antisynthetase syndrome clinical spectrum Time course. J Clin Med.

[8] Hervier B, Devilliers H, Stanciu R (2012). Hierarchical cluster and survival analyses of antisynthetase syndrome: phenotype and outcome are correlated with anti-tRNA synthetase antibody specificity. Autoimmun Rev.

[9] Mehta P, Rathore U, Naveen R (2022). Prevalent drug usage practices in adults and children with idiopathic inflammatory myopathies: registry-based analysis from the myocite cohort. J Clin Rheumatol.

[10] Oldroyd AGS, Lilleker JB, Amin T (2022). British Society for Rheumatology guideline on management of paediatric, adolescent and adult patients with idiopathic inflammatory myopathy. Rheumatology (Oxford).

[11] Lundberg IE, Fujimoto M, Vencovsky J (2021). Idiopathic inflammatory myopathies. Nat Rev Dis Primers.

[12] Leclair V, Galindo-Feria AS, Dastmalchi M (2019). Efficacy and safety of rituximab in anti-synthetase antibody positive and negative subjects with idiopathic inflammatory myopathy: a registry-based study. Rheumatology (Oxford).

[13] Krusche M, Burmester GR, Knitza J (2020). Digital crowdsourcing: unleashing its power in rheumatology. Ann Rheum Dis.

[14] Doubelt I, Springer JM, Kermani TA (2022). Self-reported data and physician-reported data in patients with eosinophilic granulomatosis with polyangiitis: comparative analysis. Interact J Med Res.

[15] Eichler GS, Cochin E, Han J (2016). Exploring concordance of patient-reported information on patientslikeme and medical claims data at the patient level. J Med Internet Res.

[16] Randell RL, Long MD, Cook SF (2014). Validation of an internet-based cohort of inflammatory bowel disease (CCFA partners). Inflamm Bowel Dis.

[17] Bhashyam A, Lubinus M, Filmore E (2022). Pain profile and opioid medication use in patients with idiopathic inflammatory myopathies. Rheumatology (Oxford).

[18] Fazal ZZ, Sen P, Joshi M (2022). COVAD survey 2 long-term outcomes: unmet need and protocol. Rheumatol Int.

[19] Kernder A, Morf H, Klemm P (2021). Digital rheumatology in the era of COVID-19: results of a national patient and physician survey. RMD Open.

[20] Springer JM, Kermani TA, Sreih A (2020). Clinical characteristics of an internet-based cohort of patient-reported diagnosis of granulomatosis with polyangiitis and microscopic polyangiitis: observational study. J Med Internet Res.

[21] Eysenbach G (2004). Improving the quality of web surveys: the checklist for reporting results of Internet E-surveys (CHERRIES). J Med Internet Res.

[22] Namsrai T, Desborough J, Chalmers A (2022). Diagnostic delay of myositis: protocol for an integrated systematic review. BMJ Open.

[23] Levi Y, Israeli-Shani L, Kuchuk M (2018). Rheumatological assessment is important for interstitial lung disease diagnosis. J Rheumatol.

[24] Gupta L, Muhammed H, Naveen R (2020). Insights into the knowledge, attitude and practices for the treatment of idiopathic inflammatory myopathy from a cross-sectional cohort survey of physicians. Rheumatol Int.

[25] Albrecht K, Huscher D, Callhoff J (2020). Trends in idiopathic inflammatory myopathies: cross-sectional data from the German national database. Rheumatol Int.

[26] Oldroyd A, Dixon W, Chinoy H (2020). Patient insights on living with idiopathic inflammatory myopathy and the limitations of disease activity measurement methods - a qualitative study. BMC Rheumatol.

[27] Rider LG, Werth VP, Huber AM et al. (2011) Measures of adult and juvenile dermatomyositis, polymyositis, and inclusion body myositis: Physician and Patient/Parent Global Activity, Manual Muscle Testing (MMT), Health Assessment Questionnaire (HAQ)/Childhood Health Assessment Questionnaire (C-HAQ), Childhood Myositis Assessment Scale (CMAS), Myositis Disease Activity Assessment Tool (MDAAT), Disease Activity Score (DAS), Short Form 36 (SF-36), Child Health Questionnaire (CHQ), physician global damage, Myositis Damage Index (MDI), Quantitative Muscle Testing (QMT), Myositis Functional Index-2 (FI-2), Myositis Activities Profile (MAP), Inclusion Body Myositis Functional Rating Scale (IBMFRS), Cutaneous Dermatomyositis Disease Area and Severity Index (CDASI), Cutaneous Assessment Tool (CAT), Dermatomyositis Skin Severity Index (DSSI), Skindex, and Dermatology Life Quality Index (DLQI). Arthritis Care Res (Hoboken) 63 Suppl 11:S118-57. 10.1002/acr.20532 10.1002/acr.20532 PMC3748930 22588740

[28] Ricci G, Fontanelli L, Torri F (2022). Fatigue as a common signature of inflammatory myopathies: clinical aspects and care. Clin Exp Rheumatol.

[29] Pawlitzki M, Acar L, Masanneck L (2022). Myositis in Germany: epidemiological insights over 15 years from 2005 to 2019. Neurol Res Pract.

[30] Pearce M, Garcia L, Abbas A (2022). Association between physical activity and risk of depression: a systematic review and meta-analysis. JAMA Psychiat.

[31] van Thillo A, Vulsteke J-B, van Assche D (2019). Physical therapy in adult inflammatory myopathy patients: a systematic review. Clin Rheumatol.

[32] Gupta L, Deshmukh P, Thornton C (2023). Addressing the unmet need for self-management strategies in idiopathic inflammatory myositis. RMD Open.

[33] Galindo-Feria AS, Horuluoglu B, Lundberg IE (2022). Anti-Jo1 autoantibodies, from clinic to the bench. Rheumatology Autoimmunity.

[34] Marie I, Hatron P-Y, Cherin P (2013). Functional outcome and prognostic factors in anti-Jo1 patients with antisynthetase syndrome. Arthritis Res Ther.

[35] Bolko L, Didier K, Salmon J, Miyara M, Toquet S, Servettaz A, Allenbach Y, Benveniste O, Hervier B (2020) Anti-Jo1 Antibody Quantification Serve as a Prognostic Factor in Anti-synthetase Syndrom [abstract]. Arthritis Rheumatol.

[36] Müller F, Boeltz S, Knitza J (2023). CD19-targeted CAR T cells in refractory antisynthetase syndrome. Lancet.

[37] Leclair V, Regardt M, Wojcik S (2016). Health-related quality of life (HRQoL) in Idiopathic inflammatory myopathy: a systematic review. PLoS One.

[38] Leclair V, Moshtaghi-Svensson J, Regardt M (2021). Distribution and trajectory of direct and indirect costs of idiopathic inflammatory myopathies. Semin Arthritis Rheum.

[39] Naveen R, Thakare DR, Agarwal V (2022). Validation of two simple patient-centered outcome measures for virtual monitoring of patients with idiopathic inflammatory myositis. Clin Rheumatol.

[40] Zarbl J, Eimer E, Gigg C (2022). Remote self-collection of capillary blood using upper arm devices for autoantibody analysis in patients with immune-mediated inflammatory rheumatic diseases. RMD Open.

[41] Saygin D, Rockette-Wagner B, Oddis C (2022). Consumer-based activity trackers in evaluation of physical activity in myositis patients. Rheumatology (Oxford).

[42] Oldroyd AGS, Krogh NS, Dixon WG (2022). Investigating characteristics of idiopathic inflammatory myopathy flares using daily symptom data collected via a smartphone app. Rheumatology (Oxford).

[43] Naveen R, Sundaram TG, Agarwal V (2021). Teleconsultation experience with the idiopathic inflammatory myopathies: a prospective observational cohort study during the COVID-19 pandemic. Rheumatol Int.

[44] Becker C, Diener M, Hueber AJ (2022). Unmet information needs of patients with rheumatic diseases: results of a cross-sectional online survey study in Germany. Int J Environ Res Public Health.

[45] Knitza J, Simon D, Lambrecht A (2020). Mobile health usage, preferences, barriers, and ehealth literacy in rheumatology: patient survey study. JMIR Mhealth Uhealth.

