# Supplementary material for: Healthcare utilization and unmet needs of patients with antisynthetase syndrome: An international patient survey
Source: Rheumatol Int. 2023 Jul 15;43(10):1925–34. doi: 10.1007/s00296-023-05372-9 (PMC10435645; doi:10.1007/s00296-023-05372-9)
Supplement: Supplementary file 1 — Supplementary file1 (DOCX 39 KB) [file 296_2023_5372_MOESM1_ESM.docx]

**Healthcare utilization and unmet needs of patients with antisynthetase syndrome: An international patient survey**

M. Weiss^1,2,^**^†^** (ORCID: 0000-0001-6406-831X), MT Holzer^3,^**^†,*^** (ORCID: 0000-0002-2064-6728), F. Muehlensiepen^4^ (ORCID: 0000-0001-8571-7286), Y. Ignatyev^4^ (ORCID: 0000-0002-3573-7980), C. Fiehn^5^ (ORCID: 0000-0001-9665-1526), J. Bauhammer^5^ (ORCID: 0000-0002-3336-0892), J. Schmidt^6,7,8^ (ORCID: 0000-0002-5589-2371), S. Schlüter^9^ (ORCID: 0000-0001-9665-1526), A. Dihkan^10^, D. Scheibner^9^, U. Schneider^11^, L. Valor Mendez^1,2^ (ORCID: 0000-0002-4872-3502), G. Corte^1,2^, L. Gupta^12,13,14^ (ORCID: 0000-0003-2753-2990), H. Chinoy^15,16^ (ORCID: 0000-0001-6492-1288), I. Lundberg^17,18^ (ORCID: 0000-0002-6068-9212), L. Cavagna^19^ (ORCID: 0000-0003-3292-1528), JHW Distler^1,2^ (ORCID: 0000-0001-7408-9333), G. Schett^1,2^ (ORCID: 0000-0001-8740-9615), J. Knitza^1,2^ (ORCID: 0000-0001-9695-0657)

Rheumatology International

Corresponding author: MT Holzer, III. Department of Internal Medicine, University Medical Center Hamburg-Eppendorf, Hamburg, Germany, m.holzer@uke.de

**Online Survey Form**

**“Health services utilization und unmet needs of patients with antisynthetase syndrome: An international patient survey”**

1. Do you agree to the above conditions? By clicking "Yes" you indicate that you would like to take part in the survey voluntarily and that you have been diagnosed with antisynthetase syndrome.

- Yes
- No

1. How did you find out about this survey?

- Treating physician
- Patient community
- Another way (please specify)

1. Which antibodies were ever found?

- Anti-Jo-1
- Anti-PL-7
- Anti-PL-12
- Anti-OJ
- Anti-EJ
- Another antibody (please specify, but only if your antibody is not included in the previous ones)

1. Where do you live?

- Germany
- Austria
- Switzerland
- Italy
- United Kingdom
- USA
- Sweden
- Spain
- France
- Other:

1. Year of birth: (please add your year of birth in a four-digit number, e.g. 1960)
2. Sex

- Female
- Male
- Diverse

1. What is your current working status?

- Full-time
- Part time
- Retired
- Inability to work
- Currently on sick leave
- Housewife/man
- Unemployed
- Completing education program
- Other (please specify):

1. If you still worked last year, how often were you on sick leave? (please add the number of days you were on sick leave last year, e.g. 25)
2. If you were on sick leave last year, what was the longest time in weeks? If you were on sick leave last year, what was the longest time in weeks? (please add the number of weeks of your longest time on sick leave last year, e.g. 25)
3. Year of diagnosis: (please specify year of diagnosis in a four-digit number, e.g. 1995)
4. Year of symptom onset: (specify year of symptom onset in a four-digit number, e.g. 1995)
5. Please check the symptoms you had at the onset:

- Joint pain
- Joint pain and swelling
- Dyspnea/cough
- "Mechanic’s hands" (rough, horny areas of the fingers, especially in the thumb and forefinger area)
- Other cutaneous lesions
- Skin ulcers
- Raynaud phenomenon (Change in finger color (white - blue - red), especially due to cold)
- Muscle strength deficit
- Muscle pain
- Fatigue
- Pain in general
- Fever

1. Please check the symptoms you ever had during disease course:

- Joint pain
- Joint pain and swelling
- Dyspnea/cough
- "Mechanic’s hands" (rough, horny areas of the fingers, especially in the thumb and forefinger area)
- Other cutaneous lesions
- Skin ulcers
- Raynaud phenomenon (Change in finger color (white - blue - red), especially due to cold)
- Muscle strength deficit
- Muscle pain
- Fatigue
- Pain in general
- Fever

1. How many specialist doctors have you seen for your symptoms until the diagnosis was made? (please specify the number of doctors seen, e.g. 5)
2. Which physicians do you regularly see for ASSD?

- Rheumatologist
- Neurologist
- Pneumologist
- Dermatologist
- Internal medicine specialist
- Immunologists
- Primary care doctor

1. Which of those is your main contact person for ASSD?

- Rheumatologist
- Neurologist
- Pneumologist
- Dermatologist
- Internal medicine specialist
- Immunologists
- Primary care doctor

1. This physician is working at a*

- University center
- Non-university center

1. During the last year, how often did you see this physician? (please specify number of times, e.g. 10)
2. Which of these tests were done to establish your diagnosis?

- Muscle biopsy
- Muscle endurance test
- Muscle strenght test
- Electromyography
- Magnetic resonance imaging (MRI)
- Muscle ultrasound
- Computer tomography (CT) scan
- Pulmonary function test
- Joint X-rays
- Joint ultrasound
- Nailfold videocapillaroscopy

1. Please check the tests you ever had during disease course:

- Muscle biopsy
- Muscle endurance test
- Muscle strength test
- Electromyography
- Magnetic resonance imaging (MRI)
- Muscle ultrasound
- Computer tomography (CT) scan
- Pulmonary function test
- Joints X-rays
- Joint ultrasound
- Nailfold videocapillaroscopy

1. What are your current and previous treatments?

- None
- Physiotherapy
- Pain medications (NSAIDs, etc.)
- Oral corticosteroids
- Intravenous corticosteroids
- Intramuscular corticosteroids
- Methotrexate
- Ciclosporin A
- Azathioprine
- Hydroxychloroquine
- Intravenous immunoglobulins
- Myophenolate mofetil
- Cyclophosphamide
- Rituximab
- TNF-alpha agents (Infliximab, Adalimumab, Etanercept, Golimumab, Certolizumab etc.)
- Plasmapheresis
- Abatacept
- Baricitinib
- Tofacitinib
- Others:

1. What are your previous treatments?

- None
- Physiotherapy
- Pain medications (NSAIDs, etc.)
- Oral corticosteroids
- Intravenous corticosteroids
- Intramuscular corticosteroids
- Methotrexate
- Ciclosporin A
- Azathioprine
- Hydroxychloroquine
- Intravenous immunoglobulins
- Myophenolate mofetil
- Cyclophosphamide
- Rituximab
- TNF-alpha agents (Infliximab, Adalimumab, Etanercept, Golimumab, Certolizumab etc.)
- Plasmapheresis
- Abatacept
- Baricitinib
- Tofacitinib
- Others:

1. Did your clinician say that you have lung involvement?

- Yes, from disease onset
- Yes, after disease onset
- No, I have no lung involvement

1. Did your clinician say that you have myositis?

- Yes, from disease onset
- Yes, after disease onset
- No, I have no myositis

1. Did your clinician say that you have arthritis?

- Yes, from disease onset
- Yes, after disease onset
- No, I have no arthritis

1. Was one of the following diseases diagnosed?

- Fibromyalgia
- Depression
- Malignancy
- None

1. How would you rate your health in general? (SF-36)

- Excellent
- Very good
- Good
- Fair
- Poor

1. Compared to one year ago, how would you rate your health in general now?

- Much better now than one year ago
- Somewhat better now than one year ago
- About the same
- Somewhat worse now than one year ago
- Much worse now than one year ago

1. Do you smoke?

- No
- Yes
- In the past

1. Would you be willing to share regularly data about your health for research purposes once a year?

- Yes
- No

1. If yes, what data would you transfer?

- Symptoms
- Life quality and well being
- Blood samples
- Blood test results
- Data from wearable (fitbit, apple watch)
- None

1. How do you rate your knowledge of your antisynthetase syndrome?

- Good
- Poor

1. How do you rate your knowledge about treatment options?

- Good
- Poor

1. Do you know useful online information sources related to antisynthetase syndrome?

- Yes - please add URL:
- No

1. Would you be interested in additional antisynthetase syndrome related information ?

- Yes
- No

1. Which of the following additional information sources related to antisynthetase syndrome would you like?

- Online written information (i.e. Wikipedia)
- Online videos
- Online community (social media/chat)
- Local community workshops
- Online healthcare discussions (HCP and patients discussion) with real time questions
- Printed medical information
- None
- Other:

1. Which of the following topics/features would you like to see on a dedicated website? (multiple)

- Treatment information
- Basic information about antisynthetase syndrome
- Contact information, list of specialized treatment and rehabilitation centers
- List of Online-Communities
- Information on clinical trials and research
- Tips for nutrition and sports
- Tips for daily disease management
- Tips for bureaucracy (insurance questions)
- Other patients’ stories
- None
