# Supplementary material for: Healthcare utilization and unmet needs of patients with antisynthetase syndrome: An international patient survey
Source: Rheumatol Int. 2023 Jul 15;43(10):1925–34. doi: 10.1007/s00296-023-05372-9 (PMC10435645; doi:10.1007/s00296-023-05372-9)
Supplement: Supplementary file 2 — Supplementary file2 (DOCX 20 KB) [file 296_2023_5372_MOESM2_ESM.docx]

**Healthcare utilization and unmet needs of patients with antisynthetase syndrome: An international patient survey**

M. Weiss^1,2,^**^†^** (ORCID: 0000-0001-6406-831X), MT Holzer^3,^**^†,*^** (ORCID: 0000-0002-2064-6728), F. Muehlensiepen^4^ (ORCID: 0000-0001-8571-7286), Y. Ignatyev^4^ (ORCID: 0000-0002-3573-7980), C. Fiehn^5^ (ORCID: 0000-0001-9665-1526), J. Bauhammer^5^ (ORCID: 0000-0002-3336-0892), J. Schmidt^6,7,8^ (ORCID: 0000-0002-5589-2371), S. Schlüter^9^ (ORCID: 0000-0001-9665-1526), A. Dihkan^10^, D. Scheibner^9^, U. Schneider^11^, L. Valor Mendez^1,2^ (ORCID: 0000-0002-4872-3502), G. Corte^1,2^, L. Gupta^12,13,14^ (ORCID: 0000-0003-2753-2990), H. Chinoy^15,16^ (ORCID: 0000-0001-6492-1288), I. Lundberg^17,18^ (ORCID: 0000-0002-6068-9212), L. Cavagna^19^ (ORCID: 0000-0003-3292-1528), JHW Distler^1,2^ (ORCID: 0000-0001-7408-9333), G. Schett^1,2^ (ORCID: 0000-0001-8740-9615), J. Knitza^1,2^ (ORCID: 0000-0001-9695-0657)

Rheumatology International

Corresponding author: MT Holzer, III. Department of Internal Medicine, University Medical Center Hamburg-Eppendorf, Hamburg, Germany, m.holzer@uke.de

| **Supplementary Table 1** Symptoms at disease onset according to antisynthetase antibody status in absolute number (percentage of subgroup in %). | | | | | | |
| --- | --- | --- | --- | --- | --- | --- |
|  | **All patients** (n=236) | **Anti-Jo-1** (n=169) | **Anti-PL-7** (n=33) | **Anti-PL-12** (n=24) | **Anti-OJ** (n=4) | **Anti-EJ** (n=5) |
| Joint Pain | 152 (53.0) | 94 (55.6) | 19 (57.6) | 11 (45.8) | 4 (100.0) | 1 (20.0) |
| Joint pain and swelling | 99 (41.9) | 86 (50.9) | 7 (21.2) | 8 (33.3) | 2 (50.0) | 0 (0) |
| Dyspnea/cough | 120 (50.8) | 78 (46.2) | 19 (57.6) | 15 (62.5) | 3 (75.0) | 5 (100.0) |
| Mechanic’s hands | 107 (45.3) | 80 (47.3) | 16 (48.5) | 10 (41.7) | 2 (50.0) | 2 (40.0) |
| Other cutaneous lesions | 35 (14.8) | 16 (9.5) | 10 (30.3) | 8 (33.3) | 2 (50.0) | 1 (20.0) |
| Skin ulcers | 5 (2.1) | 3 (1.8) | 0 (0) | 1 (4.2) | 0 (0) | 0 (0) |
| Raynaud‘s phenomenon | 91 (38.6) | 58 (34.3) | 19 (57.6) | 9 (37.5) | 1 (25.0) | 2 (40.0) |
| Muscle strength deficit | 117 (49.6) | 89 (52.7) | 13 (9.4) | 8 (33.3) | 1 (25.0) | 1 (20.0) |
| Muscle pain | 130 (55.1) | 95 (56.2) | 14 (42.4) | 15 (62.5) | 2 (50.0) | 3 (60.0) |
| Fatigue | 159 (67.4) | 109 (64.5) | 21 (63.6) | 20 (83.3) | 3 (75.0) | 5 (100.0) |
| Pain in general | 79 (33.5) | 47 (27.8) | 9 (27.3) | 5 (20.8) | 2 (50.0) | 1 (20.0) |
| Fever | 69 (29.2) | 68 (40.2) | 10 (30.3) | 10 (41.7) | 3 (75.0) | 2 (40.0) |

| **Supplementary Table 2** Physician confirmed lung involvement, myositis and arthritis at disease onset according to antibody in absolute number (percentage of subgroup in %). | | | | | | |
| --- | --- | --- | --- | --- | --- | --- |
|  | **All patients** (n=236) | **Anti-Jo-1** (n=169) | **Anti-PL-7** (n=33) | **Anti-PL-12** (n=24) | **Anti-OJ** (n=4) | **Anti-EJ** (n=5) |
| Lung involvement | 160 (67.8) | 116 (68.6) | 21 (63.6) | 18 (75.0) | 3 (75.0) | 5 (100.0) |
| Myositis | 135 (57.2) | 101 (59.8) | 22 (66.7) | 9 (37.5) | 2 (50.0) | 3 (60.0) |
| Arthritis | 78 (33.1) | 67 (39.6) | 6 (18.2) | 1 (4.2) | 1 (25.0) | 0 (0) |
| Complete triad | 42 (17.8) | 38 (22.5) | 3 (9.1) | 0 (0) | 0 (0) | 0 (0) |

| **Supplementary Table 3** Physicians reported as the main contact person for ASSD. N=236 for all patients. | |
| --- | --- |
| **Physician** | N (percentage of all patients) |
| Rheumatologist | 187 (79.2) |
| Pneumologist | 28 (11.9) |
| Primary care doctor | 7 (3.0) |
| Immunologist | 5 (2.1) |
| Internal medicine specialist | 5 (2.1) |
| Neurologist | 4 (1.7) |

| **Supplementary Table 4** Physicians reported to be seen regularly by patients. N=236 for all patients. | |
| --- | --- |
| **Physician** | N (percentage of all patients in %) |
| Rheumatologist | 216 (91.5) |
| Pneumologist | 127 (53.8) |
| Primary care doctor | 124 (52.5) |
| Dermatologist | 36 (15.3) |
| Internal medicine specialist | 36 (15.3) |
| Neurologist | 21 (8.9) |
| Immunologist | 12 (5.1) |

| **Supplementary Table 5** Reported links to useful online information regarding ASSD. Sorted alphabetically. | |
| --- | --- |
| **Number** | **URL** |
| 1 | www.antisynthetase.org |
| 2 | www.dermnetz.org |
| 3 | www.DVASS.org |
| 4 | Facebook groups: antisynthetase support group, Jo-1 facebook group, myositis group |
| 5 | www.hopkinsrheumatology.org/rheumtv/antisynthetase-disease-overview-johns-hopkins/ass-featuredimage-diagnos/ |
| 6 | www.internetmedicin.se/behandlingsoversikter/reumatologi/idiopatiska-inflammatoriska-myopatier/ |
| 7 | www.myositis.org |
| 8 | www.myositis-netz.de/myositis/myositis-bei-overlap-syndrom |
| 9 | www.myosit.reumatiker.se |
| 10 | www.nih.gov |
| 11 | www.orphanet.net |
| 12 | www.rheuma-badenbaden.de |
| 13 | www.rheuma-liga.de |
| 14 | www.rheumatiker.se |
| 15 | www.understandingmyositis.org/explaining-myositis |
| 16 | www.webmd.com |
| Other information used: | Medical journals, myositis experts (Lorenzo Cavagna, Rohit Aggarwal) |
